# Establishment of a novel CNV-related prognostic signature predicting prognosis in patients with breast cancer

**DOI:** 10.1186/s13048-021-00823-y

**Published:** 2021-08-08

**Authors:** Wei Hu, Mingyue Li, Qi Zhang, Chuan Liu, Xinmei Wang, Jing Li, Shusheng Qiu, Liang Li

**Affiliations:** 1 Department of Thyroid and Breast Surgery, Zibo Central Hospital, Zibo, 255036 China; 2 Department of Rehabilitation Medicine, The Third Affilated Hospital, Sun Yat-sen University, Guangzhou, 510000 China; 3 Blood Transfusion Department, Zibo Central Hospital, Zibo, 255036 China; 4 Department of Pathology, ZiBo Central Hospital, Zibo, 255036 China

**Keywords:** Copy number variation, Breast cancer, Gene signature, TCGA, Prognosis, Bioinformatics

## Abstract

**Background:**

Copy number variation (CNVs) is a key factor in breast cancer development. This study determined prognostic molecular characteristics to predict breast cancer through performing a comprehensive analysis of copy number and gene expression data.

**Methods:**

Breast cancer expression profiles, CNV and complete information from The Cancer Genome Atlas (TCGA) dataset were collected. Gene Expression Omnibus (GEO) chip data sets (GSE20685 and GSE31448) containing breast cancer samples were used as external validation sets. Univariate survival COX analysis, multivariate survival COX analysis, least absolute shrinkage and selection operator (LASSO), Chi square, Kaplan-Meier (KM) survival curve and receiver operating characteristic (ROC) analysis were applied to build a gene signature model and assess its performance.

**Results:**

A total of 649 CNV related-differentially expressed gene obtained from TCGA-breast cancer dataset were related to several cancer pathways and functions. A prognostic gene sets with 9 genes were developed to stratify patients into high-risk and low-risk groups, and its prognostic performance was verified in two independent patient cohorts (*n* = 327, 246). The result uncovered that 9-gene signature could independently predict breast cancer prognosis. Lower mutation of PIK3CA and higher mutation of TP53 and CDH1 were found in samples with high-risk score compared with samples with low-risk score. Patients in the high-risk group showed higher immune score, malignant clinical features than those in the low-risk group. The 9-gene signature developed in this study achieved a higher AUC.

**Conclusion:**

The current research established a 5-CNV gene signature to evaluate prognosis of breast cancer patients, which may innovate clinical application of prognostic assessment.

**Supplementary Information:**

The online version contains supplementary material available at 10.1186/s13048-021-00823-y.

## Introduction

Copy number variations (CNVs), which are DNA fragments with varied copy number from 1 kb to several Mb in the human genome, include DNA fragment deletions, insertions, duplications, and compound multipoint variants [1]. CNVs are often present in various types of tumors, and are currently considered as a key factor in genetic variation of tumors [2–5]. CNVs at multiple sites in the genome can cause heterogeneity of the genome and molecular phenotype, leading to the occurrence and development of complex diseases including cancers [2, 6, 7]. Ding et al. reported the diversity of genomes of patients with primary breast cancer that are manifested as frequent gene rearrangements and copy number changes [8]. Shlien et al. used gene chips to compare 770 normal genomes, and found that 49 oncogenes were surrounded by CNV [9]. Stolz et al. demonstrated that about 50% of lung cancer patients show cell cycle-checkpoint kinase 2 gene (CHEK2) inactivation [10].

According to data released by the American Cancer Society in 2018, breast cancer is the most common malignancy among women worldwide and the second leading cause of cancer-related death to women with high [11]. In recent decades, the incidence of breast cancer in China is increasing and is showing a younger trend, noticeably, breast cancer has become a malignant tumor with the highest incidence among Chinese women [12, 13]. The causes of breast cancer are highly complex [14]. In recent decades, great progress has been made in the diagnosis, surgery, chemotherapy and molecular therapy of breast cancer, but the prognosis of breast cancer is still unsatisfactory due to its high heterogeneity and complexity. Therefore, the biological molecular mechanism of breast cancer development should be further studied and explored.

In this study, we examined the correlation between CNV-associated gene expression profiles and clinical outcomes in 1069 breast cancer patients recorded in the Cancer Genome Atlas (TCGA). CNV-associated genes were used to develop a prognostic model for the prediction of overall survival (OS) of breast cancer patients. The results of this study may provide a strategy targeting autophagy for predicting and monitoring the prognosis of breast cancer patients.

## Material and methods

### Microarray data profile

**Fig. 1:**
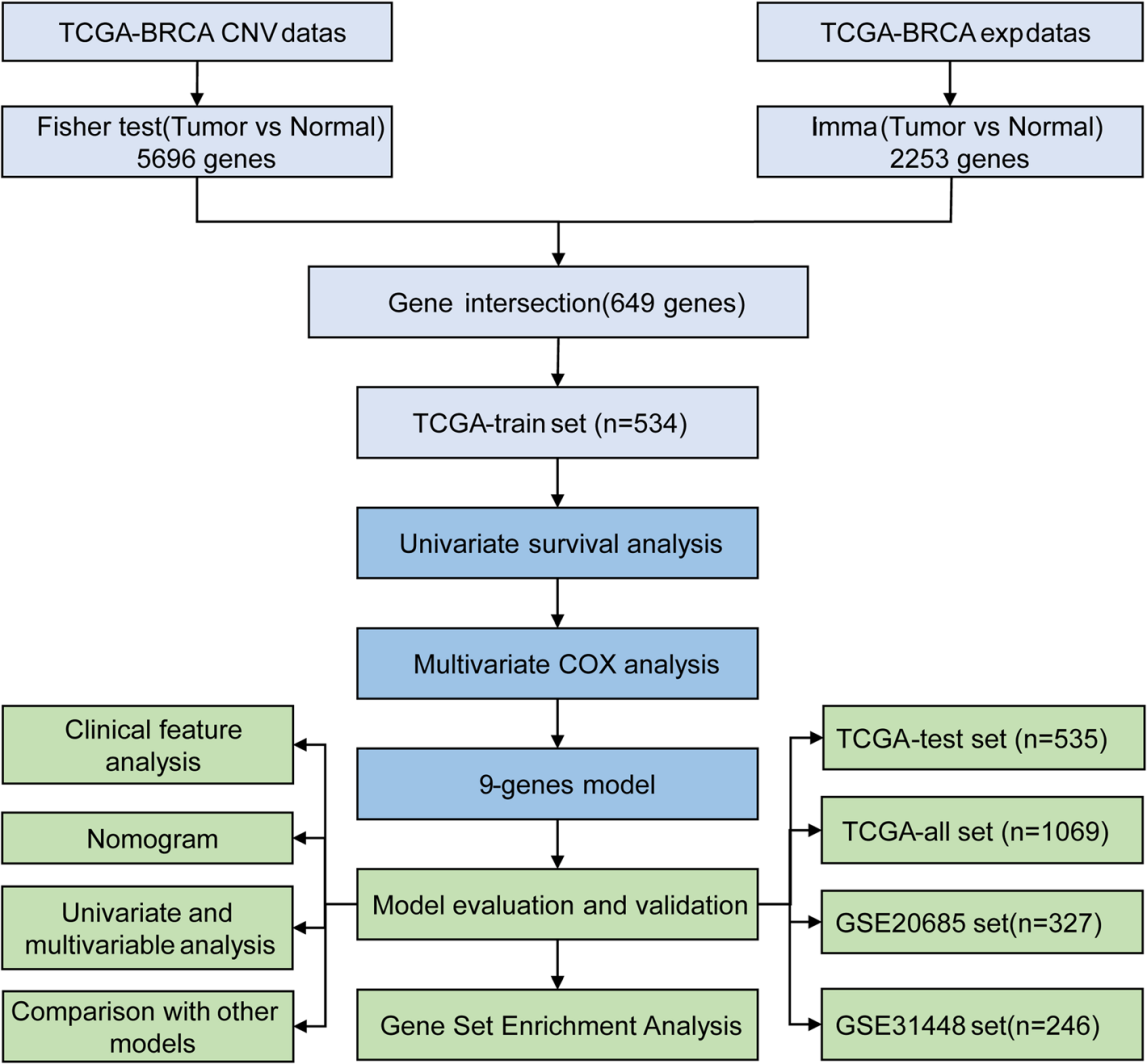
Work flow chart

The study design is shown in Fig. 1. Gene expression profile and CNV dataset TCGA [15] with complete follow-up information were obtained on June 30, 2020, 1069 tumor samples with integral clinical information were obtained and randomly classified into the training cohort (*n* = 534), the testing cohort (*n* = 536). The two groups were similar in age distribution, sex, follow-up time, and proportion of death. After clustering the gene expression profiles of the two data sets, the number of samples of dichotomy was similar.

The GSE20685 [16] and GSE31448 [17] chip data sets with survival time of 327 and 246 samples were downloaded from Gene Expression Omnibus (GEO) (http://www.ncbi.nlm.nih.gov/geo/) on June 30, 2020. The clinical information of the three data groups is shown in Table 1.

**Table 1:** Sample clinical information for three data sets

Clinical Features	TCGA-BRCA	GSE20685	GSE31448
**Event**			
0	921	244	167
1	148	83	79
**T Stage**			
T1	279	101	
T2	617	188	
T3	132	26	
T4	38	12	
TX	3	0	
**N Stage**			
N0	502	137	
N1	357	87	
N2	120	63	
N3	73	40	
NX	17	0	
**M Stage**			
M0	884	319	
M1	22	8	
MX	163	0	
**Stage**			
I	181		
II	606		
III	240		
IV	20		
X	22		
**ER Status**			
Negative	235		
Positive	786		
Unknown	48		
**PR Status**			
Negative	335		
Positive	683		
Unknown	51		
**Her2 Status**			
Negative	549		
Positive	159		
Unknown	361		
**Age**			
≤ 60	598	282	
> 60	471	45	
**Subtype**			
Basal	187		72
Her2	77		22
LumA	552		85
LumB	202		42
Normal	39		25
Unknown	12		0

### Identification tumor-specific CNV and differentially expressed genes (DEGs)

The chromosome segments in the CNV segment file were matched to genes using bedTools [18], and only the mean value of CNV cells with absolute value greater than 0.2 were kept for further analysis. The difference of CNV identification between tumor samples and normal samples was determined by chi-square test (FDR < 0.05).

The DEGs between tumor and normal samples were calculated using the Limma package [19], and the threshold filter was FDR < 0.01 and |log2FC| > 1.

After drawing the Wayne map of the differentially expressed CNV and the DEGs, 649 common genes were found.

### Functional enrichment

Gene ontology (GO) and Kyoto Encyclopedia of Genes and Genomes (KEGG) were used to analyzed correlation biological functions and pathways of DEG using WebGestaltR (v0.4.2) [20] in R package.

### Identification of prognostic CNV-related genes

Univariate Cox regression, least absolute shrinkage and selection operator (LASSO) regression and multivariate Cox regression analyses were employed to explore the performance of CNV-related genes in predicting OS of breast cancer. Genes were determined as potential prognostic genes when *p* value was < 0.05 in Univariate Cox regression analysis. LASSO-penalized and multivariate analysis were next performed for further screening. Hazard ratios (HRs) and regression coefficient were calculated for each gene, and 9 CNV-related genes were ultimately included.

### Construction of prognostic gene signature

The risk-score model for prognosis prediction of breast cancer patients was the combination of each optimal prognostic CNV-related gene expression level multiplying relative regression coefficient weight calculated from the multivariate model according to the following formula:

$$ \mathrm{RiskScore}=\sum \mathrm{iCoefficient}\left(\mathrm{mRNAi}\right)\times \mathrm{Expression}\left(\mathrm{mRNAi}\right) $$

All patients in the training cohort were classified into low- and high-risk groups according to the median of risk scores. The Kaplan–Meier survival curves of both groups were plotted, and the receiver operating characteristic (ROC) curve for OS prediction was used to assess the specificity and sensitivity of the model.

### Validation of gene signature

Risk score of the patients in TCGA testing cohort, entire TCGA cohort, GSE20685 and GSE31448 dataset were calculated, and patients were assigned into the high-risk and low-risk group with the cut-off value calculated from the training cohort. The Kaplan–Meier survival curves of both groups were plotted, and the ROC curve for OS prediction was used to assess the specificity and sensitivity of the model.

### Analysis of clinical feature, mutation gene and immune score

Analysis of RiskScore in clinical feature including T, N, M, Stage, Age were analyzed. Mutation annotation format (MAF) files were processed and visualized by R package maftools [21]. StromalScore, ImmuneScore and ESTIMATEScore were analyzed using ESTIMATE [22] in package.

### Comparison with published models

By referring to the literature, we selected three prognostic risk models (10-gene signature (Huang) [23], 4-gene signature (Qi) [24], 19-gene signature (Su) [25] and 6-gene signature (Wang) [26]) for comparison with our 9-gene model, and evaluated them by KM curve, receiver operating characteristic (ROC) curve.

## Results

### Genes with CNV and expression differences were screened

**Fig. 2:**
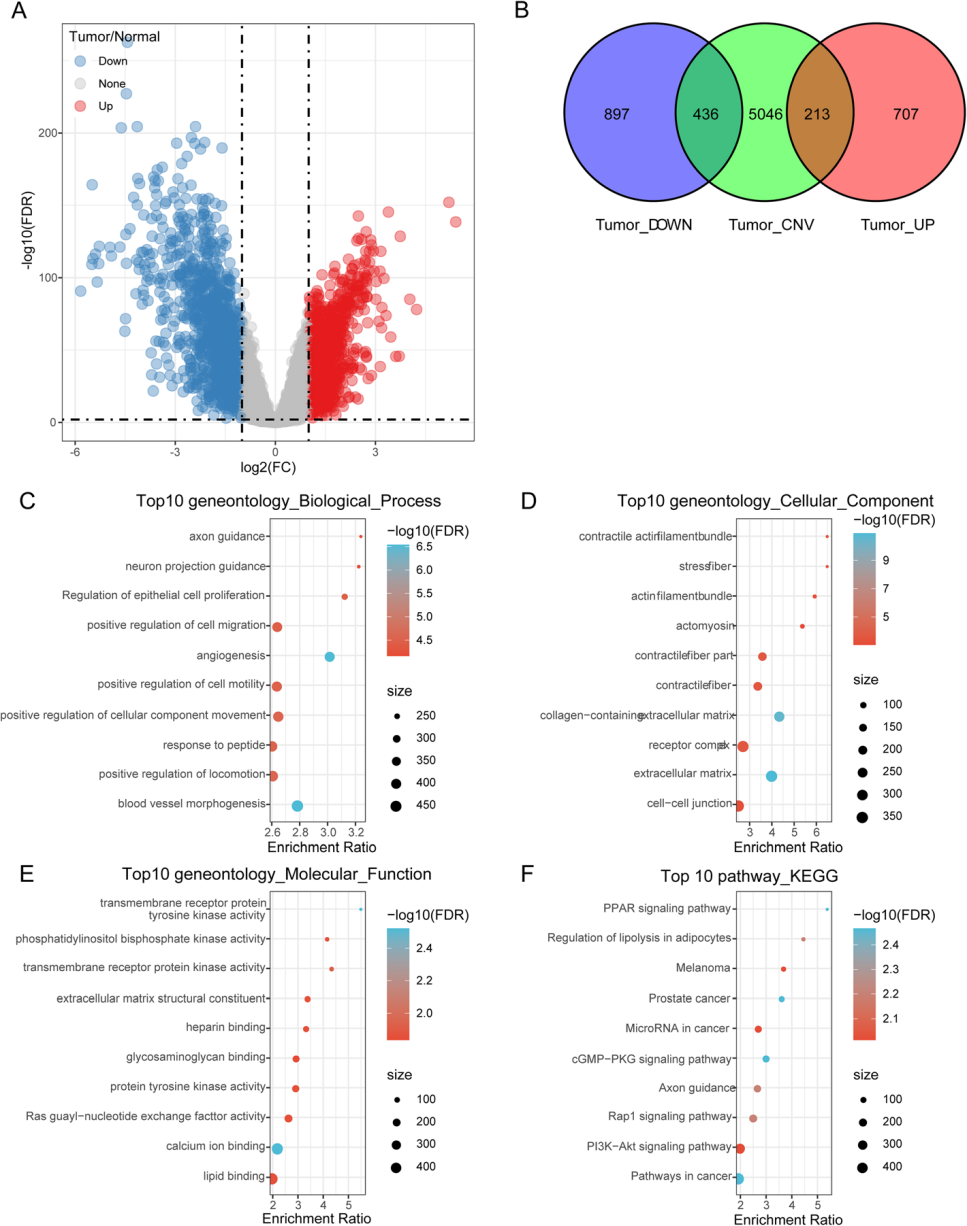
Genes with CNV and expression differences were screened. **a**: Volcano map of differentially expressed genes between Tumor and Normal. **b**: Venn diagram of specific CNV and differentially expressed genes. **c**: BP annotation map of differentially expressed genes. **d**: CC annotation map of differentially expressed genes. **e**: MF annotation map of differentially expressed genes. **f**: KEGG annotation map of differentially expressed genes

Bedtools was used to detect CNV genes related to breast cancer progression, here we screened 5696 significant differential CNV gene between breast cancer sample and normal sample. Under the condition of FDR < 0.01 and |log2FC| > 1, 920 up-regulation genes and 1333 down-regulation genes were obtained between breast cancer sample and normal sample (Fig. 2A) using Limma package. Venn diagram analysis showed that there were 649 genes with CNV and expression differences (Fig. 2B). KEGG and GO analyses conducted to explore the potential mechanism of these DEGs revealed that DEGs were mainly enriched in positive regulation of cell motility in biological process, cell-cell junction in cellular component and lipid binding in molecular function (Fig. 2C-E). Moreover, KEGG analysis demonstrated that those genes mainly were involved in PPAR signaling pathway, prostate cancer, Rap1 signaling pathway, PI3K-Akt signaling pathway and other pathways in cancer (Fig. 2F).

### Establishment of CNV related genes prognostic model

**Fig. 3:**
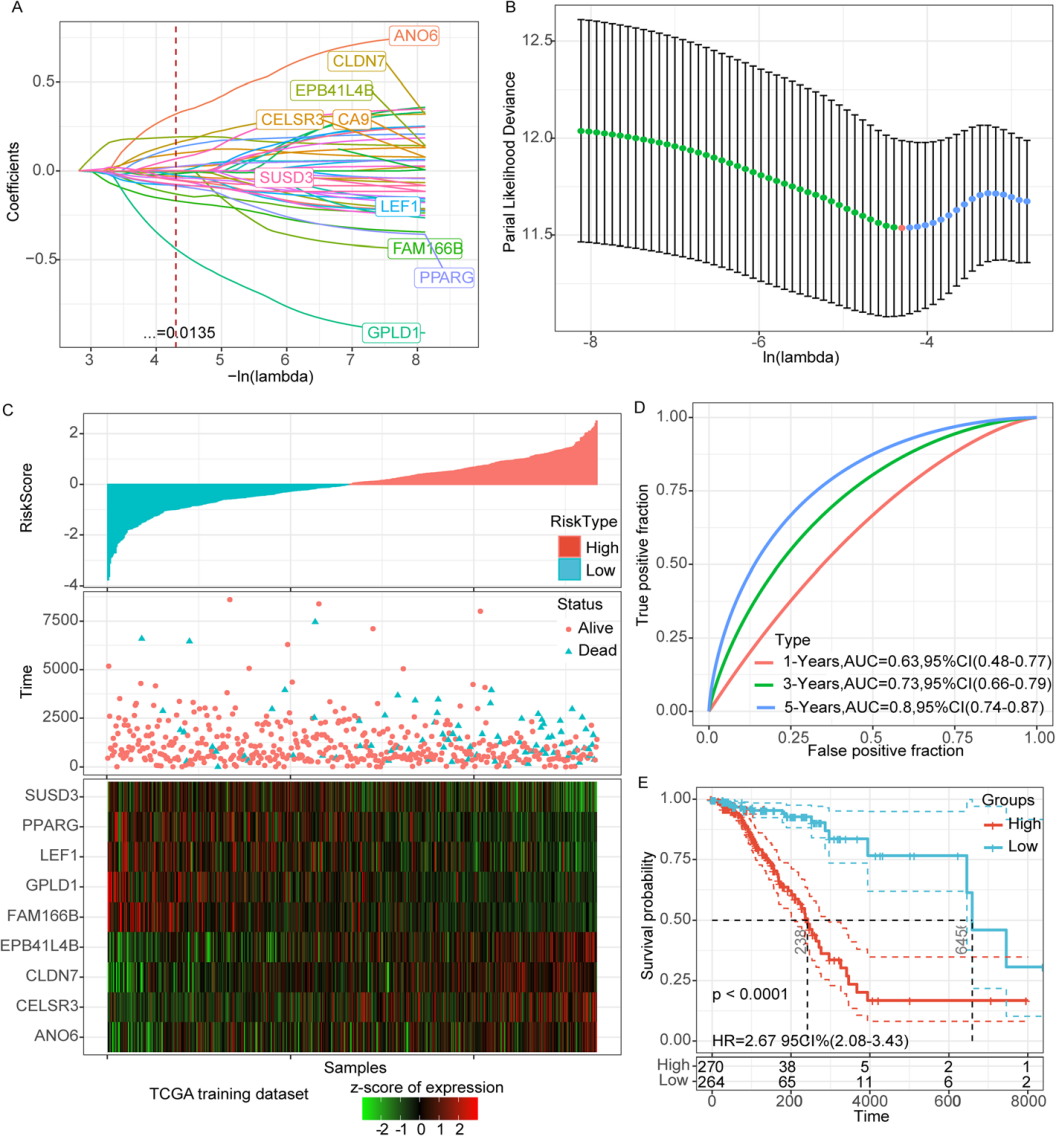
Establishment of CNV related genes prognostic model. **a**: The change trajectory of each independent variable, the horizontal axis represents the log value of the independent variable lambda, and the vertical axis represents the coefficient of the independent variable. **b**: The confidence interval under each lambda. **c**: RiskScore distribution, survival time and survival status and 9-gene expression in the TCGA training set. **d**: ROC curve and AUC of 9-gene signature in the TCGA training set. **e**: KM survival curve of 9-gene signature in the TCGA training set

Base on TCGA training dataset, above 649 genes were subjected to univariate Cox survival analysis, and screened 39 DEG. A prognostic signature was developed to predict breast cancer patients’ overall survival. Based on the expression profile of the TCGA training dataset, LASSO Cox regression and multivariate Cox regression analyses were performed (Fig. 3A, B). A prognostic model was constructed based on ANO6, CELSR3, CLDN7, EPB41L4B, FAM166B, GPLD1, LEF1, PPARG and SUSD3. The risk score of breast cancer prognosis was determined with the following formula: RiskScore = 0.629*ANO6 + 0.147*CELSR3 + 0.381*CLDN7+ 0.273*EPB41L4B-0.357*FAM166B -0.843*GPLD1–0.202*LEF1–0.202*PPARG -0.127*SUSD3. KM survival analysis showed that apart from CLDN7 and GPLD1, other genes could accurately divide samples into higher and lower-risk group (Figure S1).

The median level of the risk score was used to classify the breast cancer patients in TCGA training dataset into low- and high-risk groups. For the risk score and survival status calculated by the prognostic model and the heatmap of 9 genes, see Fig. 3C. Time-dependent ROC analysis demonstrated that AUC for 1-, 3-, 5-year survival was 0.63, 0.73, 0.8, respectively (Fig. 3D). KM survival analysis showed that the survival rate of the patients in the low-risk group was significantly higher than that in the high-risk group (*p* < 0.0001) (Fig. 3E).

### Validation of the risk score in TCGA test set and all TCGA dataset

**Fig. 4:**
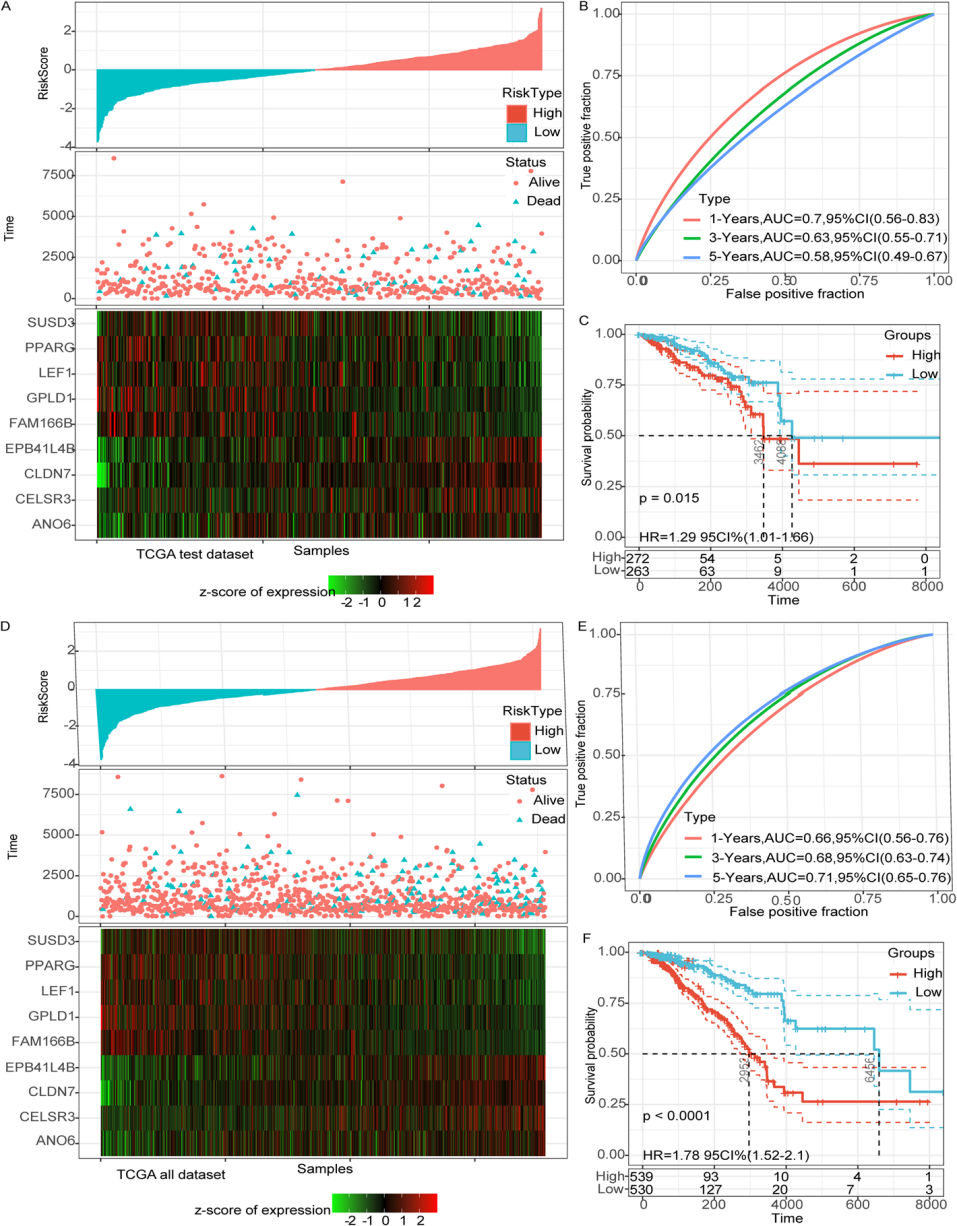
Validation of the risk score in TCGA test set and all TCGA dataset. **a**: RiskScore distribution, survival time and survival status and 9-gene expression in the TCGA test set. **b**: ROC curve and AUC of 9-gene signature in the TCGA test set. **c**: KM survival curve of 9-gene signature in the TCGA test set. **d**: RiskScore distribution, survival time and survival status and 9-gene expression in the TCGA all data sets. **e**: ROC curve and AUC of 9-gene signature in the TCGA all data sets. **f**: KM survival curve of 9-gene signature in the TCGA all data sets

In order to verify the robustness of the model, the same coefficient to the training set was used, and the model was applied to the TCGA validation dataset and entire dataset. The risk score of each sample was determined according to the expression level of the sample, and the RiskScore distribution and sample survival status was drew (Fig. 4A, D). Time-dependent ROC analysis demonstrated that AUC for 1-, 3-, 5-year survival was 0.7, 0.63, 0.58, respectively in TCGA test dataset, and 0.66, 0.69 and 0.71 respectively in all TCGA dataset (Fig. 4B, E). KM survival analysis showed that the survival rate of the patients in the low-risk group was significantly higher than that in the high-risk group in both TCGA test dataset (*p* = 0.015) and all TCGA dataset (*p* < 0.0001) (Fig. 4C, F).

### Validation of the risk score in GSE20685 and GSE31448

**Fig. 5:**
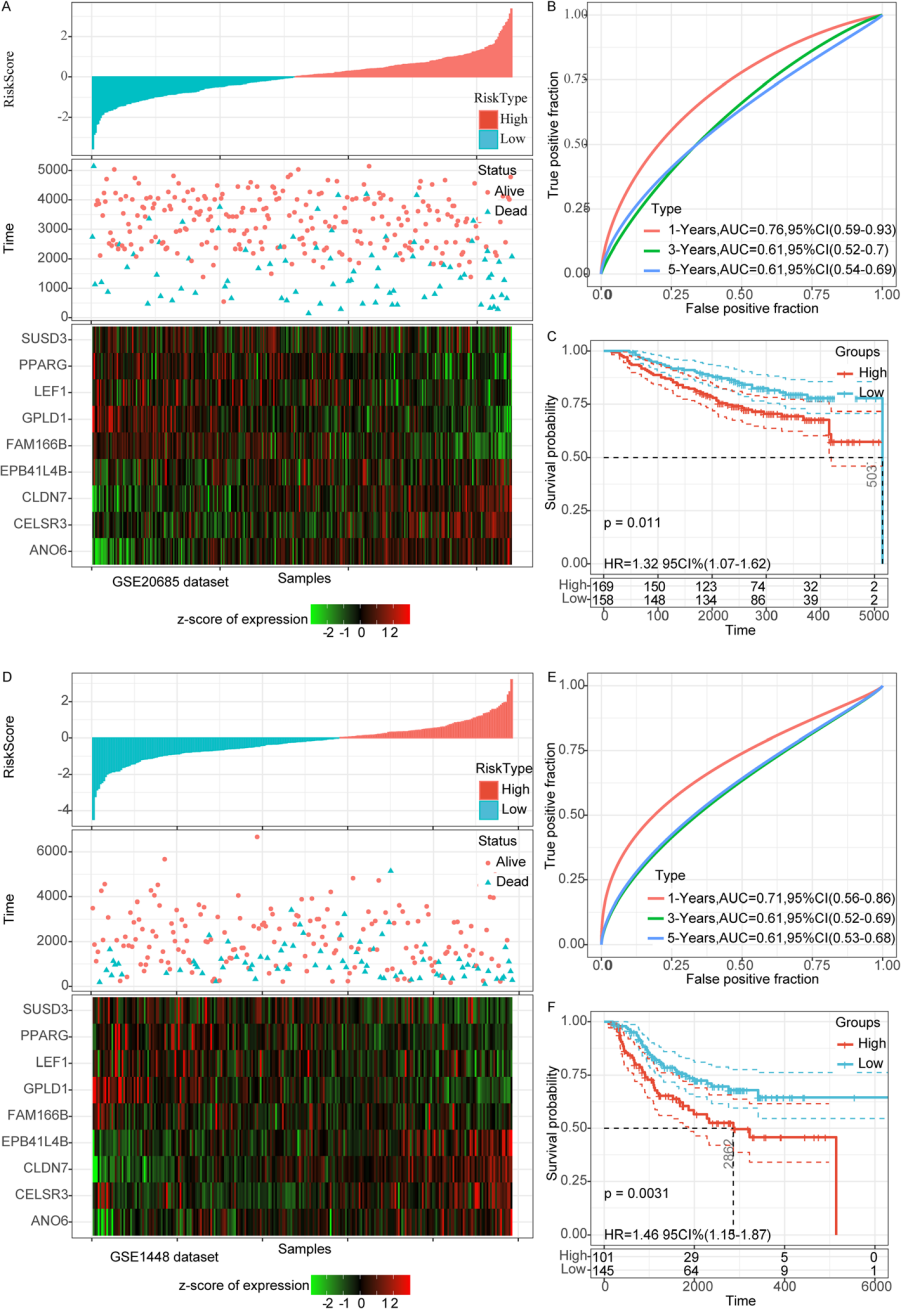
Validation of the risk score in GSE20685 and GSE31448. **a**: RiskScore distribution, survival time and survival status and 9-gene expression in the GSE20685 data set. **b**: ROC curve and AUC of 9-gene signature in the GSE20685 data set. **c**: KM survival curve of 9-gene signature in the GSE20685 data set. **d**: RiskScore distribution, survival time and survival status and 9-gene expression in the GSE31448 data set. **e**: ROC curve and AUC of 9-gene signature in the GSE31448 data set. **f**: KM survival curve of 9-gene signature in the GSE31448 data set

To determine cross-platform applicability, we applied the model to the GSE20685 and GSE31448 datasets with the same coefficients as the training set to calculate the risk score of each sample according to the expression of the model gene, and drew the RiskScore distribution (Fig. 5A, D). Time-dependent ROC analysis demonstrated that AUC for 1-, 3-, 5-year survival was 0.78, 0.61 and 0.61, respectively in GSE20685 dataset, and 0.71, 0.61 and 0.61 in GSE31448 dataset (Fig. 5B, E). KM survival analysis showed that the survival rate of the patients in the low-risk group was significantly higher than that in the high-risk group in both GSE20685 dataset (*p* = 0.011) and GSE31448dataset (*p* = 0.0031) (Fig. 5C, F).

### Comparison of clinical characteristics between high and low risk groups

**Fig. 6:**
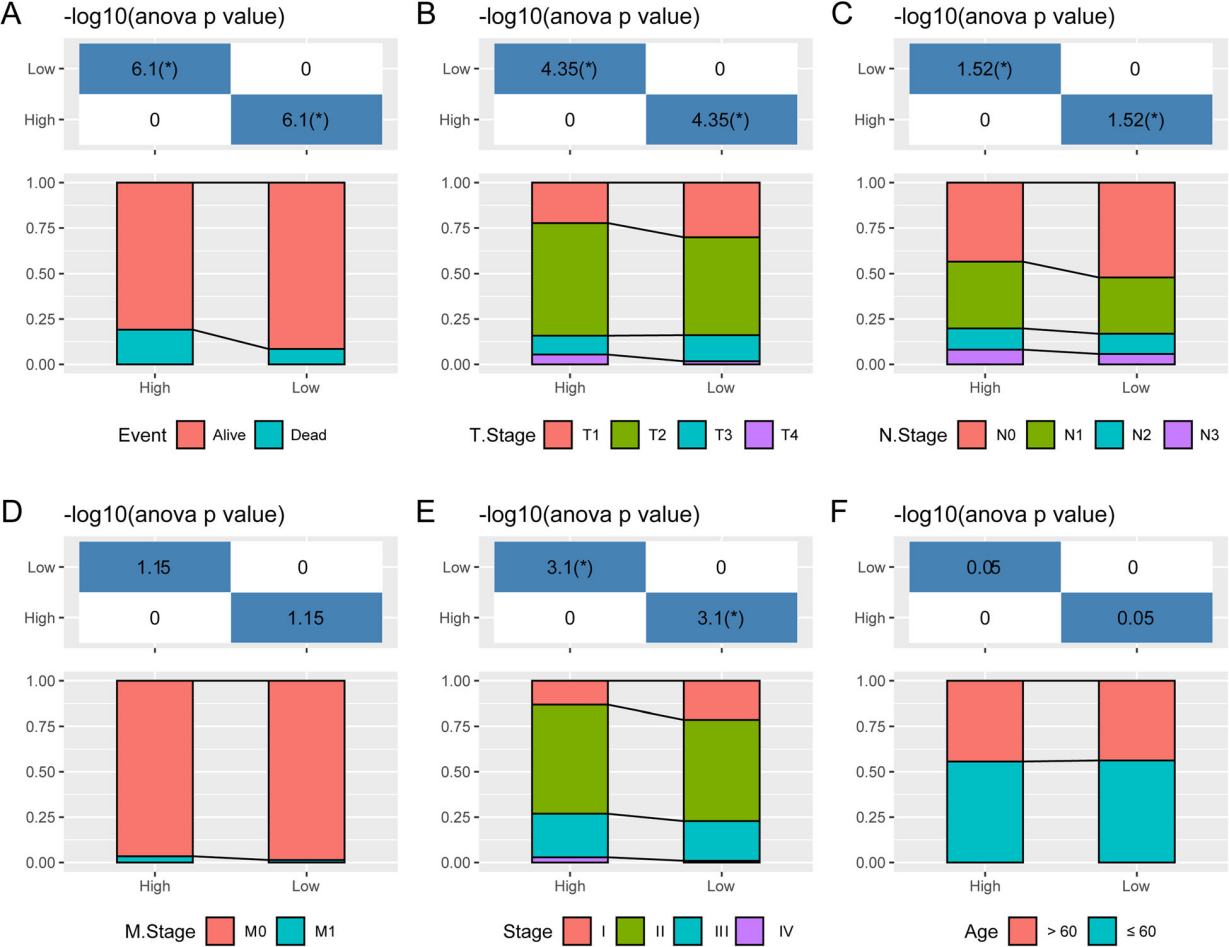
Comparison of clinical characteristics between high and low risk groups. **a**: Distribution of Alive and Dead sample between high and low risk groups in TCGA dataset. **b**: Distribution of T stage sample between high and low risk groups in TCGA dataset. **c**: Distribution of N stage sample between high and low risk groups in TCGA dataset. **d**: Distribution of M stage sample between high and low risk groups in TCGA dataset. **e**: Distribution of Stage stage sample between high and low risk groups in TCGA dataset. **f**: Distribution of Age sample between high and low risk groups in TCGA dataset

In the TCGA dataset, the distribution of clinical features in the high- and low- risk subgroups were compared. Results showed that there were more samples with a high-risk clinical features in high-risk group, such as T2, T3, and T4, higher degree of differentiation of N1 and N2 and N3, Stage II, III and IV (Fig. 6).

### Comparison of molecular mutation and immune score between high- and low-risk groups

**Fig. 7:**
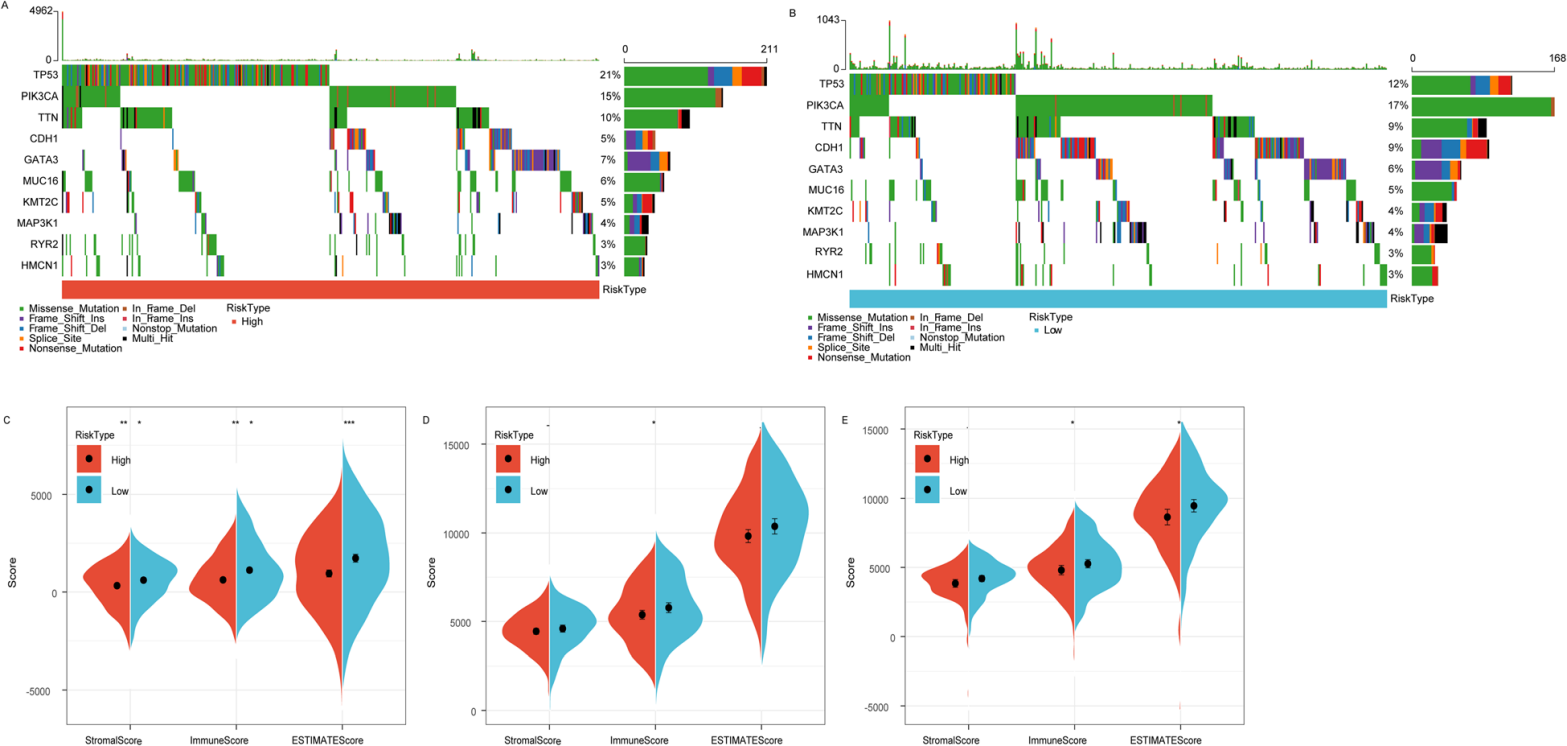
Comparison of molecular mutation and immune score between high and low risk groups. **a**: Distribution of molecular mutations in high risk groups in the TCGA dataset. **b**: Distribution of molecular mutations in low risk groups in the TCGA dataset. **c**: Comparison of immune scores between high and low risk groups in TCGA dataset. **d**: Comparison of immune scores between high and low risk groups in GSE20685 dataset. **e**: Comparison of immune scores between high and low risk groups in GSE31448 dataset

In the TCGA dataset, we compared the distribution of mutation frequencies across high- and low-risk groups, and found that TP53 mutation frequencies were higher, and CDH1 and PIK3CA mutation frequencies were lower in the high-risk group (Fig. 7A-B).

To examine the relationship of immune scores between high- and low-risk groups of the TCGA dataset, GSE20685 and GSE31448 datasets, the R software package ESTIMATE was used to assess StromalScore, ImmuneScore, ESTIMATEScore. The results showed that the three immune scores were higher in the low-risk group than those in the high-risk group (Fig. 7C-E).

### Analysis of clinical characteristics in RiskScore

**Fig. 8:**
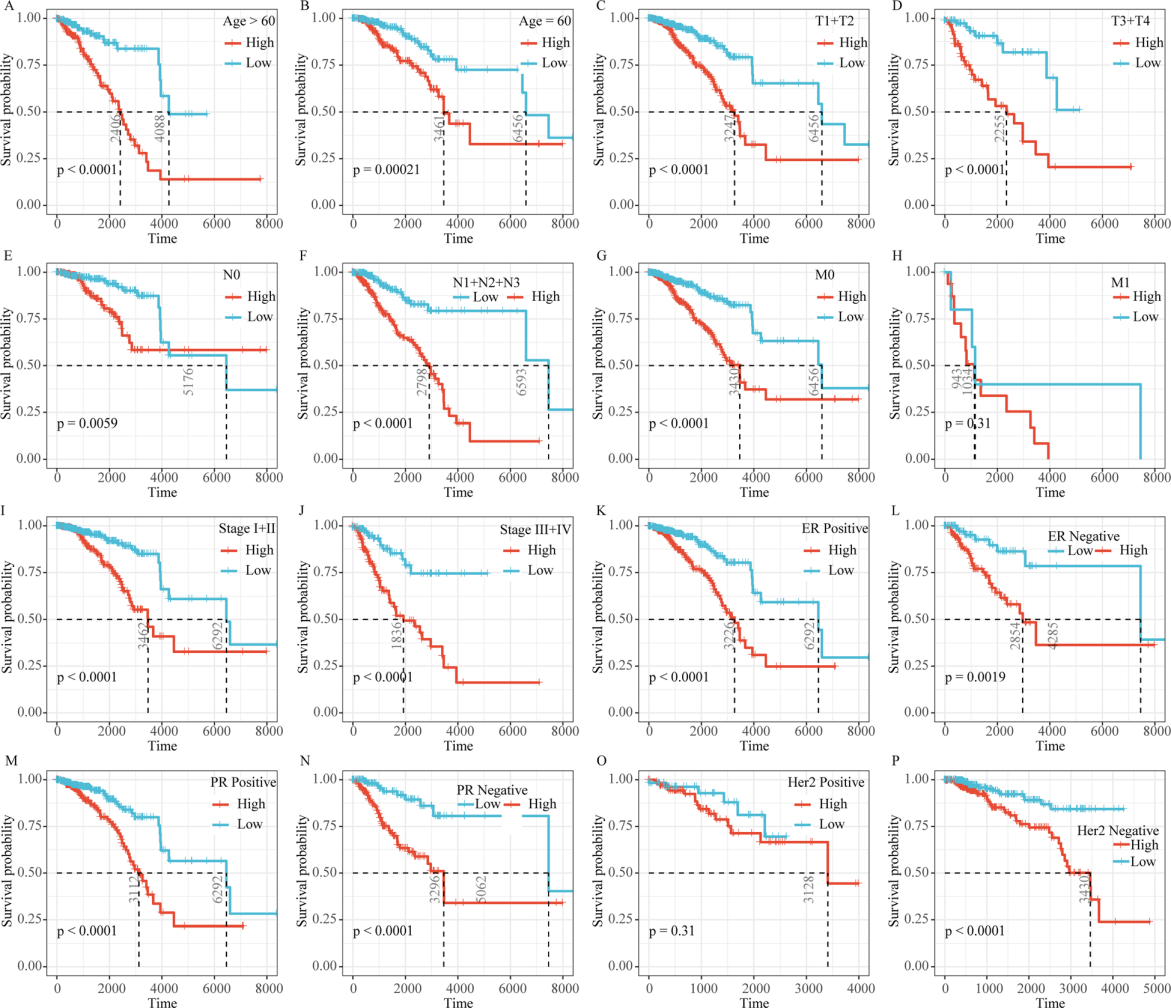
The performance of the risk model on clinical features of the TCGA data set

RiskScore analysis in clinical features showed that 9-gene signature could significantly distinguish high- and low-risk groups by age, T Stage, N Stage, M0 Stage, Stage, ER status, PR status and HER2 status in TCGA dataset (Fig. 8), but M1 Stage and Her2 positive could not effectively distinguish high and low risk groups. This further indicated that our model still had a strong predictive ability in different clinical signs.

**Fig. 9:**
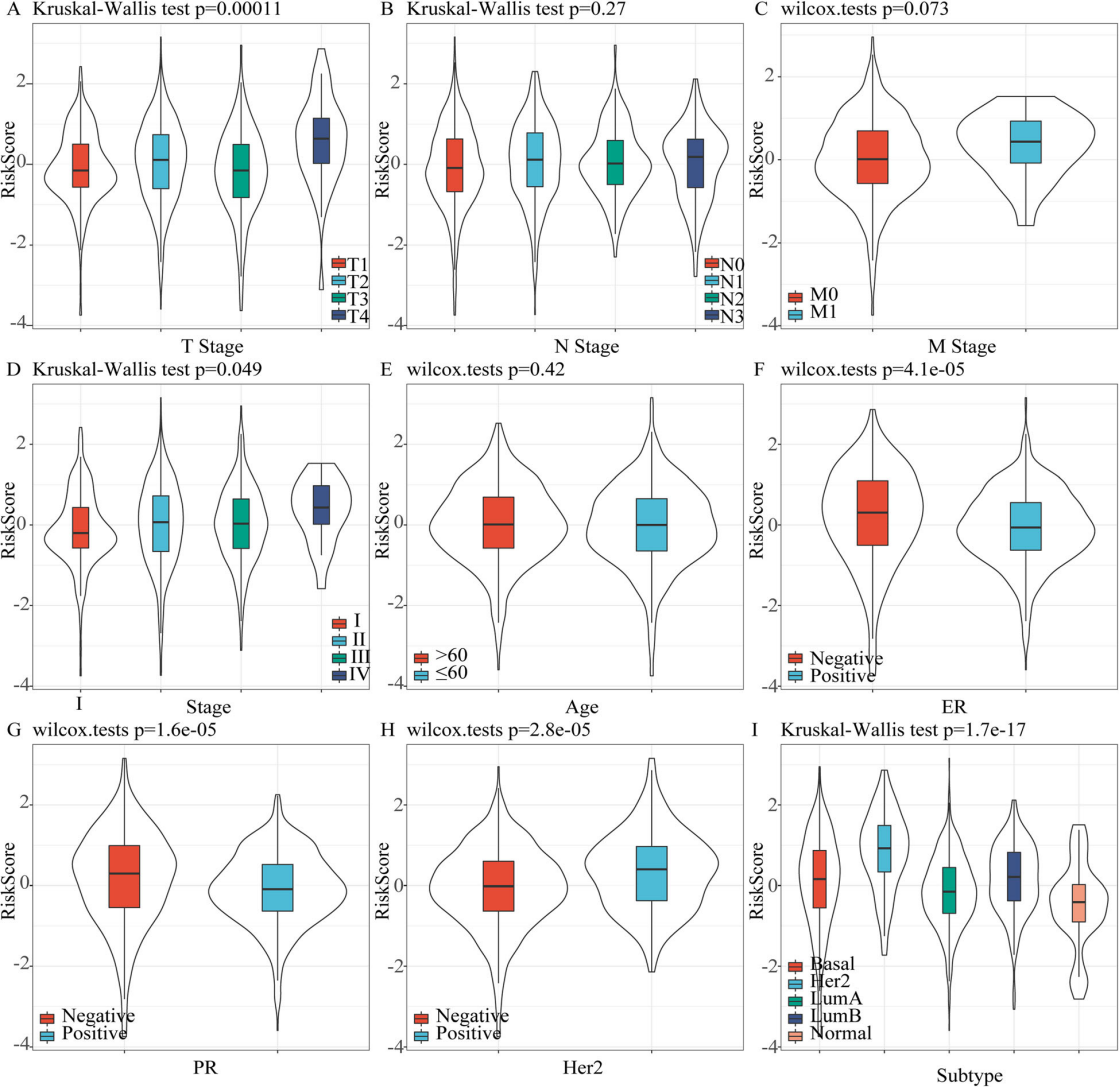
The distribution of RiskScore in different clinical characteristics and molecular subtypes in TCGA dataset

By comparing the distribution of RiskScore between groups of clinical features, we found that there were significant differences between groups of T Stage, Stage, ER status, PR status, HER2 status and molecular subtypes (*p* < 0.05) (Fig. 9).

### Independence of RiskScore

**Fig. 10:**
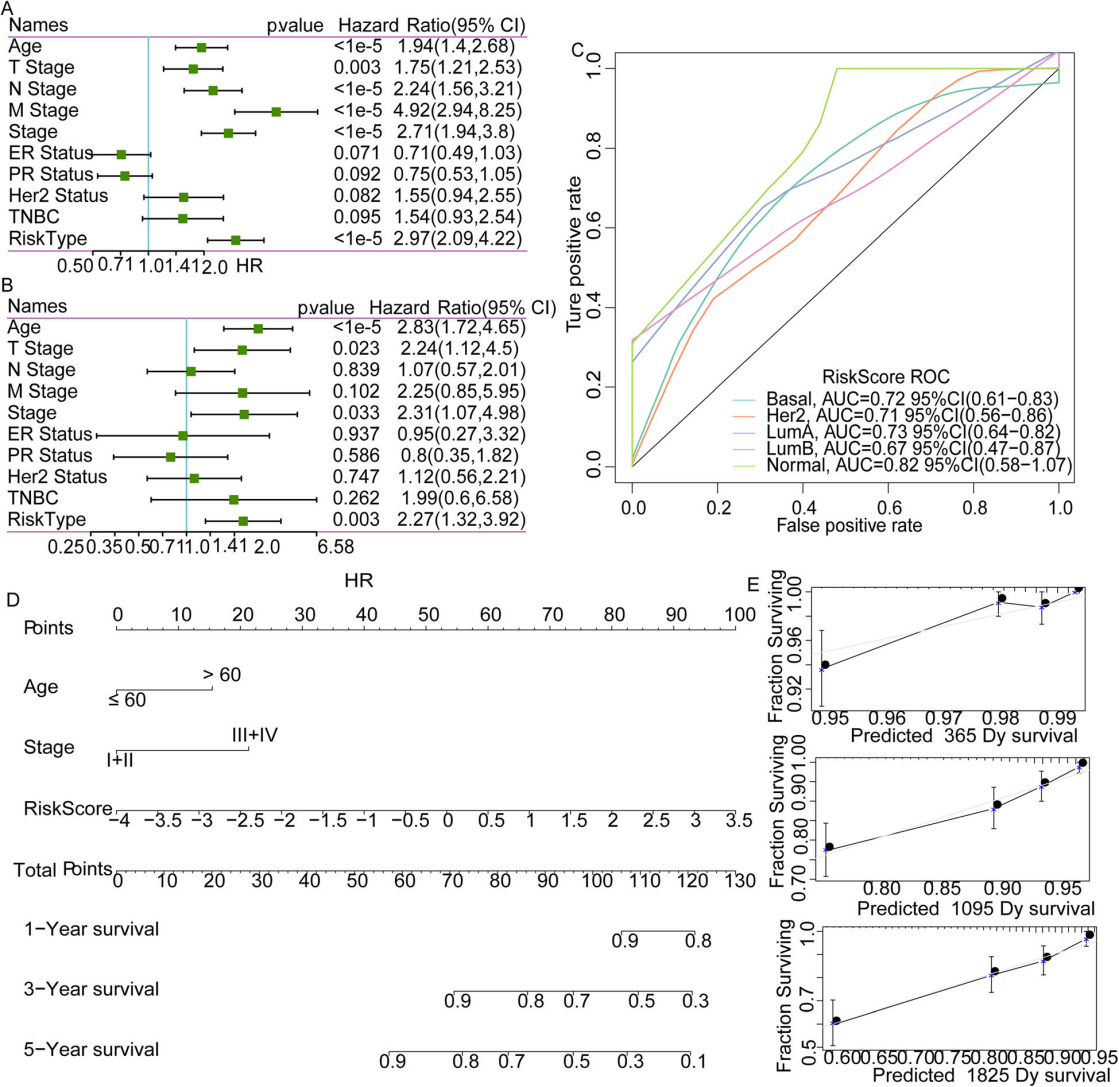
Independence of RiskScore. **a**: Univariate Cox survival analysis of clinical characteristics and RiskScore. **b**: Multivariate Cox survival analysis of clinical characteristics and RiskScore. **c**: Nomogram constructed by RiskScore and Clinical characteristics. **d**: Corrected plot of survival rates in nomogram

To assess whether the model was an independent predictor of breast cancer, univariate and multivariate analyses were performed on clinical factors and RiskScore. The results showed that showed independent prognostic power of Age, T Stage, Stage and RiskScore (Fig. 10A, B). We used clinical features Age, Stage, and RiskScore together to build a nomogram model using TCGA dataset. The results demonstrated that the RiskScore feature had the greatest influence on the survival prediction, indicating that the risk model based on the 9 genes can better predict patients’ prognosis (Fig. 10C). In addition, we also visualized the prediction performance of the nomogram data for 1-, 3- and 5-year survival (Fig. 10D), and the data proved that the nomogram had a strong prediction performance.

### Advances of the model

**Fig. 11:**
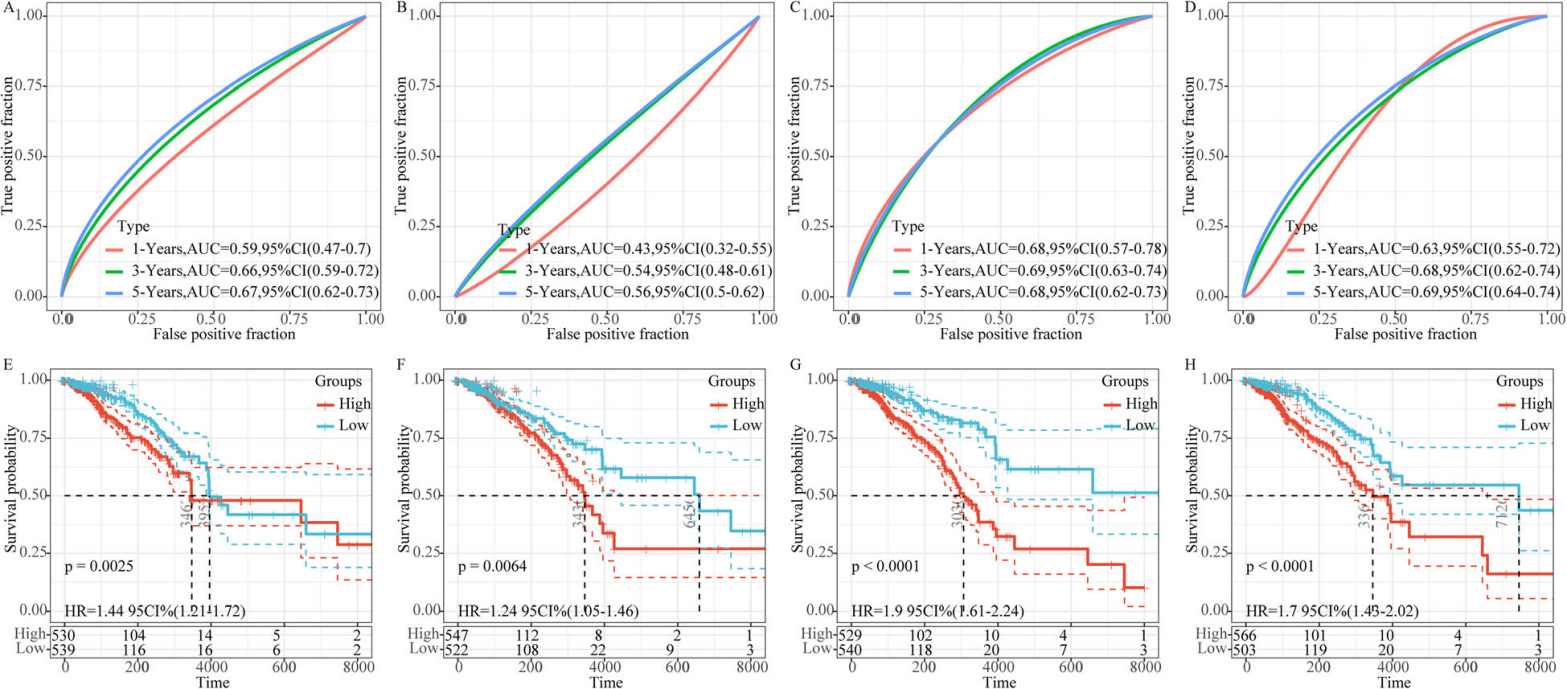
Superiority of the model. **a**, **e**: ROC and KM curves of 10-gene signature (Huang) risk model; **b**, **F**: ROC and KM curves of 4-gene signature (Qi). **c**, **G**: ROC and KM curves of 19-gene signature (Su). **d**, **H**:ROC and KM curves of 6-gene signature (Wang)

By consulting the literature, we further selected four prognostic-related risk models (a 10-gene signature (Huang), a 4-gene signature (Qi), a 19-gene signature (Su) and a 6-gene signature (Wang)) for comparison with our 9-gene model. In order to promote the comparability of the models, we calculated the risk scores of each BRCA sample in TCGA using the same method based on the corresponding genes in the four models, and divided the samples into the high-risk group and the low-risk group. The ROC curves of the four models showed that except for the 1-, 3-, and 5-year AUC of the 19-gene signature (Su) model, which are close to our model, the AUC of other three model were all lower than our model (Fig. 11A-D). KM curves indicated that the BRCA prognosis in the high- and low-risk group samples were different (log rank *p* < 0.05) (Fig. 11E-H).

## Discussion

A total of 5696 CNV-related genes and 2253 DEGs were acquired from TCGA-BRCA dataset. After the intersection, 649 CNV-associated DEGs were determined and subjected to univariate survival analysis, multivariate COX analysis and LASSO regression analysis to construct a prognostic model. Finally, 9 CNV-related prognostic genes (ANO6, CELSR3, CLDN7, EPB41L4B, FAM166B, GPLD1, LEF1, PPARG and SUSD3) model was developed. After a comprehensive analysis of the clinical information, we found that these 9 genes were associated with multiple clinical features of breast cancer.

After reviewing the existing literature, in addition to tumor-associated mutations, researchers have also focused on other variant subtypes such as copy number variation [27]. Several pathological CNVs, such as CNV of BRCA1, MTUS1 and hTERT, have been identified in the initiation and progression of breast cancer subtypes, suggesting a specific contribution of CNVs to breast cancer [6, 28]. The CNV signature has the potential to be an effective biomarker for differentiating different tumors. However, considering that CNVs are widely distributed in tumor genomes, traditional experimental methods based on gene microarrays and real-time PCR to identify specific CNV patterns for specific tumor subtypes are often inefficient and time-consuming. In this aspect, tumor-specific CNVs could be used as a new tool to identify specific breast cancer-associated CNVs based on whole-genome sequencing data. Thus, copy number correlation studies may open a new direction to breast cancer treatment and prognosis. Several copy number-related prognostic indicators have been proposed. The CNV map of the MammaPrint™ gene or Oncotype DX® gene could predict the prognosis of patients with breast cancer [29, 30]. This study identified prognostic genes associated with CNV based on the whole genome sequence of breast cancer from the TCGA dataset, which may be provided new diagnostic indicators.

By reviewing the existing literature, we found that these 9 genes were more or less associated with tumor development. ANO6 has a higher expression in gliomas, and inhibition ANO6 suppresses the proliferation and invasion of gliomas cells [31]. The significance of ANO6 has also been found in bleeding disorders [32] and bone dysplasia [33]. CELSR3 mRNA expression is upregulated in hepatocellular carcinoma and indicates poor prognosis [34, 35]. Claudin-7 (CLDN7) is aberrantly expressed in some types of cancers including gastric cancer [36], human clear cell renal cell carcinoma [37] and colorectal cancer [38]. EPB41L4B is upregulated in prostate adenocarcinoma [39]. Knockout and suppression therapies designed for LEF1 have been shown to be effective in reducing tumor growth, migration, and invasion of CLL, CRC, glioblastoma multiforme (GBM), and renal cell carcinoma (RCC) [40]. PPARG promotes the differentiation of bladder epithelial cells and regulates the expression of mitochondrial genes [41]. A study has shown that a lack of SUSD3 expression in breast cancer tissues may be an important predictor of non-response to aromatase inhibitors [42]. However, FAM166B and GPLD1 have not been thoroughly studied in tumors.

Somatic mutation analysis of samples from the high- and low-risk groups indicated that differences in mutated genes may account for the genetic differences in breast cancer patients. The mutation of TP53 and TTN was higher, and PIK3R1 was lower in the high-risk group than in the low-risk group. Interestingly, these three genes have been shown to have some tumor suppressive effects in previous studies [43–45].

The advance of this study lies in the discovery that copy number variation is associated with the mechanism of breast cancer, which opens a new direction for breast cancer treatment. Also, we identified hub genes closely associated with breast cancer survival. Most of these genes have been shown to affect tumor progression and have the potential to be used in targeted therapies. However, most of the genes have not been well studied in relation to breast cancer.

This study found that copy number variants are associated with breast cancer and screened hub genes on copy number variants, which may become new targets for breast cancer treatment.

## Supplementary Information

**Additional file 1.**

## Data Availability

The analyzed data sets generated during the study are available from the corresponding author on reasonable request.
