# Supplementary figures and images for: Establishment of a novel CNV-related prognostic signature predicting prognosis in patients with breast cancer
Source: J Ovarian Res. 2021 Aug 8;14:103. doi: 10.1186/s13048-021-00823-y (PMC8349487; doi:10.1186/s13048-021-00823-y)

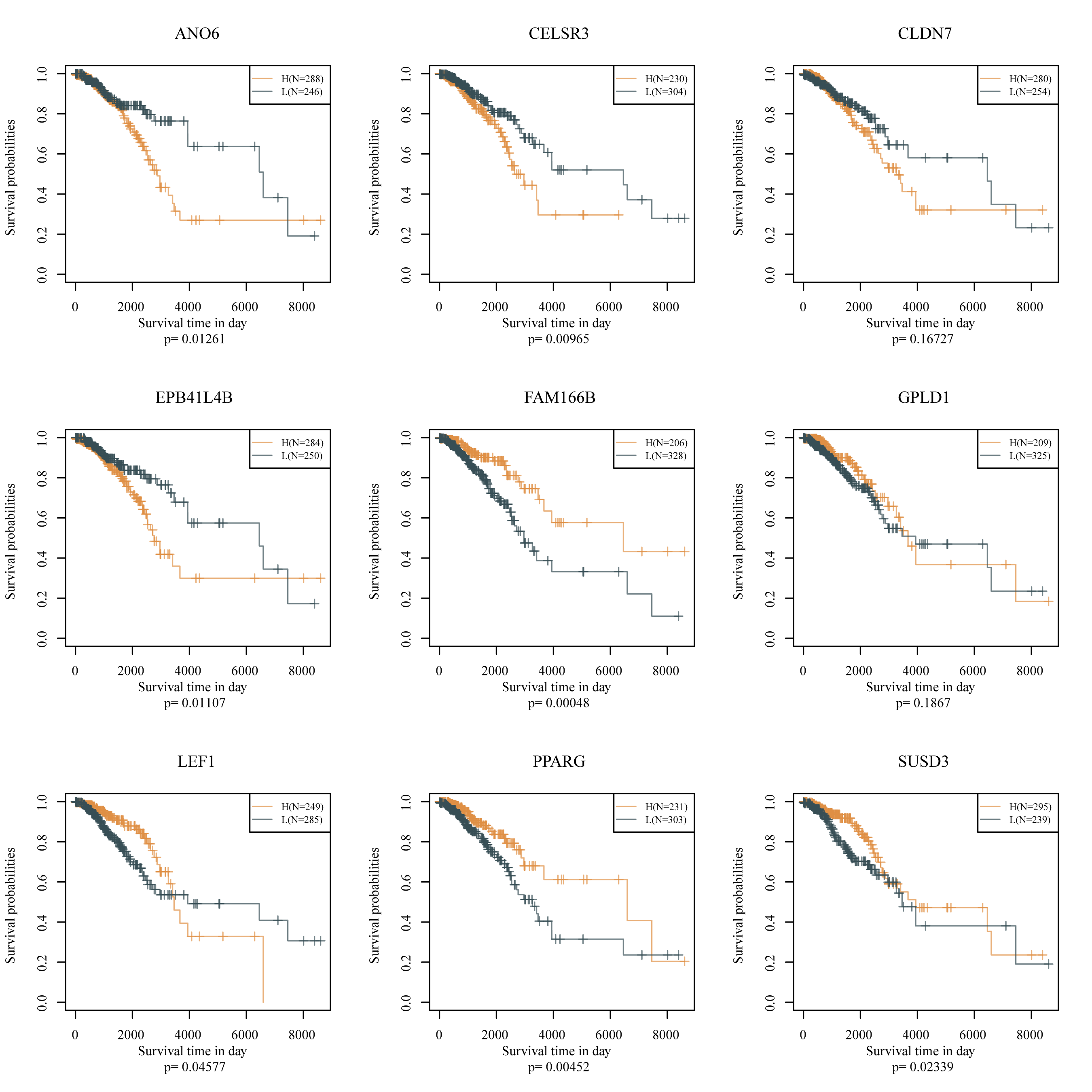

Supplement: Supplementary file 1 — Additional file 1. [file 13048_2021_823_MOESM1_ESM.tif]
